# High performance micro-flow cytometer based on optical fibres

**DOI:** 10.1038/s41598-017-05843-7

**Published:** 2017-07-17

**Authors:** S. Etcheverry, A. Faridi, H. Ramachandraiah, T. Kumar, W. Margulis, F. Laurell, A. Russom

**Affiliations:** 1 Department of Applied Physics, KTH Royal Institute of Technology, Stockholm, Sweden; 2 Department of Fibre Optics, RISE Acreo AB, Stockholm, Sweden; 3 Division of Proteomics and Nanobiotechnology, Science for Life Laboratory, KTH Royal Institute of Technology, Solna, Sweden

## Abstract

Flow cytometry is currently the gold standard for analysis of cells in the medical laboratory and biomedical research. Fuelled by the need of point-of-care diagnosis, a significant effort has been made to miniaturize and reduce cost of flow cytometers. However, despite recent advances, current microsystems remain less versatile and much slower than their large-scale counterparts. In this work, an all-silica fibre microflow cytometer is presented that measures fluorescence and scattering from particles and cells. It integrates cell transport in circular capillaries and light delivery by optical fibres. Single-stream cell focusing is performed by Elasto-inertial microfluidics to guarantee accurate and sensitive detection. The capability of this technique is extended to high flow rates (up to 800 µl/min), enabling a throughput of 2500 particles/s. The robust, portable and low-cost system described here could be the basis for a point-of-care flow cytometer with a performance comparable to commercial systems.

## Introduction

Flow cytometry is a powerful technique for the analysis of cells and the diagnosis of health disorder^1^. Typically, flow cytometers integrate fluidics, optics and electronics^2^. The fluidic system organizes fluorescently labelled cells into a single stream (i.e. cell focusing) by means of a sheath flow, and leads them to a detection chamber. The optical system uses laser beams to target the cells flowing through the detection chamber, where scattered and fluorescent light is measured. Even though flow cytometers provide good sensitivity and impressive throughput of thousands of cells per second, commercial systems are bulky, costly and require trained personnel for operation and maintenance. This has limited their use to the central laboratory and core facilities. To bring such devices to point-of-care (POC) applications there is a need for miniaturisation, ruggedness, portability, and cost reduction. Microflow cytometers that combine microfluidics and miniaturized detection systems are a promising solution for POC diagnosis^3^. The lab-on-a-chip platform has been used to develop such systems during the last years. This platform has allowed the integration of light delivery, using for instance embedded optical fibre^4–7^ or slab waveguides^8–11^ into microfluidic channels, where cells are transported and focused. However, despite the innovative ideas demonstrated, current integrated systems are less versatile or slower than conventional flow cytometers.

Polymer-based materials, such as embossed thermoplastics^9^ and elastomers^12^ are commonly employed to define the fluidics and waveguides in microfluidics-based lab-on-a-chip. Nevertheless, polymer microchips are not optimal due to misalignment when the device is pressurized, and polymer auto-fluorescence at short wavelength excitation^13^. In contrast, silica is an excellent material for laser light handling, for being inert, and for keeping its shape under high pressure. It has been extensively used for optical fibres and capillary tubes, which are fabricated reproducibly in kilometre length. Silica optical fibres and capillaries can be assembled using equipment developed for optical communication to obtain low-cost optofluidic devices^14^ without the need for expensive manufacturing instrumentation or clean-room facilities. These advantages could be exploited in building a silica fibre-based flow cytometer. In order for such a device to be competitive, it would have to provide sensitive and accurate analysis of cells at high throughput, which can be achieved by integrating single-stream particle focusing into a suitable optical and fluidic design.

A significant effort is put into developing efficient single-stream focusing mechanism in microchannels. This is accomplished, for instance, by multi-layer sheath-flow^15^, acoustophoresis^16–18^ and inertial microfluidics^19–23^. The capability of these methods for flow cytometry has been demonstrated^15, 18–22^, but the systems remain bulky, relying on external microscopy. Recently, elasto-inertial microfluidics was introduced as a passive and simple alternative for focusing cells^24–33^. It exploits fluid inertia and elastic forces that appear when cells flow in a viscoelastic fluid made using an elasticity enhancer, which in most cases consists of the polymers polyethylene oxide (PEO) or polyvinylpyrrolidone (PVP). Elasto-inertial microfluidics has the unique advantage of providing single-stream cell focusing in straight channels^24–30^ without the need of external fields or specially designed microchips. This approach simplifies single-stream focusing and facilitates its integration into miniaturized optical systems, but to date focusing in viscoelastic fluids using PEO or PVP has only been demonstrated at low Reynolds numbers (Re < 1), preventing its use for cell counting applications.

In this work, a fully integrated all-silica fibre microflow cytometer is presented. It consists of a circular capillary for the transport of fluorescently labelled cells to an integrated micro-chamber. Elasto-inertial microfluidics is used to focus the cells at the centre of the capillary. Light is delivered to the micro-chamber through an optical fibre. Fluorescence and scattered light is collected with the same fibre, whereby the cells can be identified^34^. In contrast to previous work, stable single-stream focusing in PEO fluids is achieved at high Reynolds numbers and high flow rates, enabling accurate high-throughput cytometry.

In the experiments below, elasto-inertial focusing is optimized for particles of different sizes, enabling efficient optical excitation and detection. Subsequently, the fibre microflow cytometer is validated, by counting and classifying fluorescent particles and cancer cells through laser induced fluorescence and back scattering.

## Results

### Optofluidic component and detection principle

**Figure 1:**
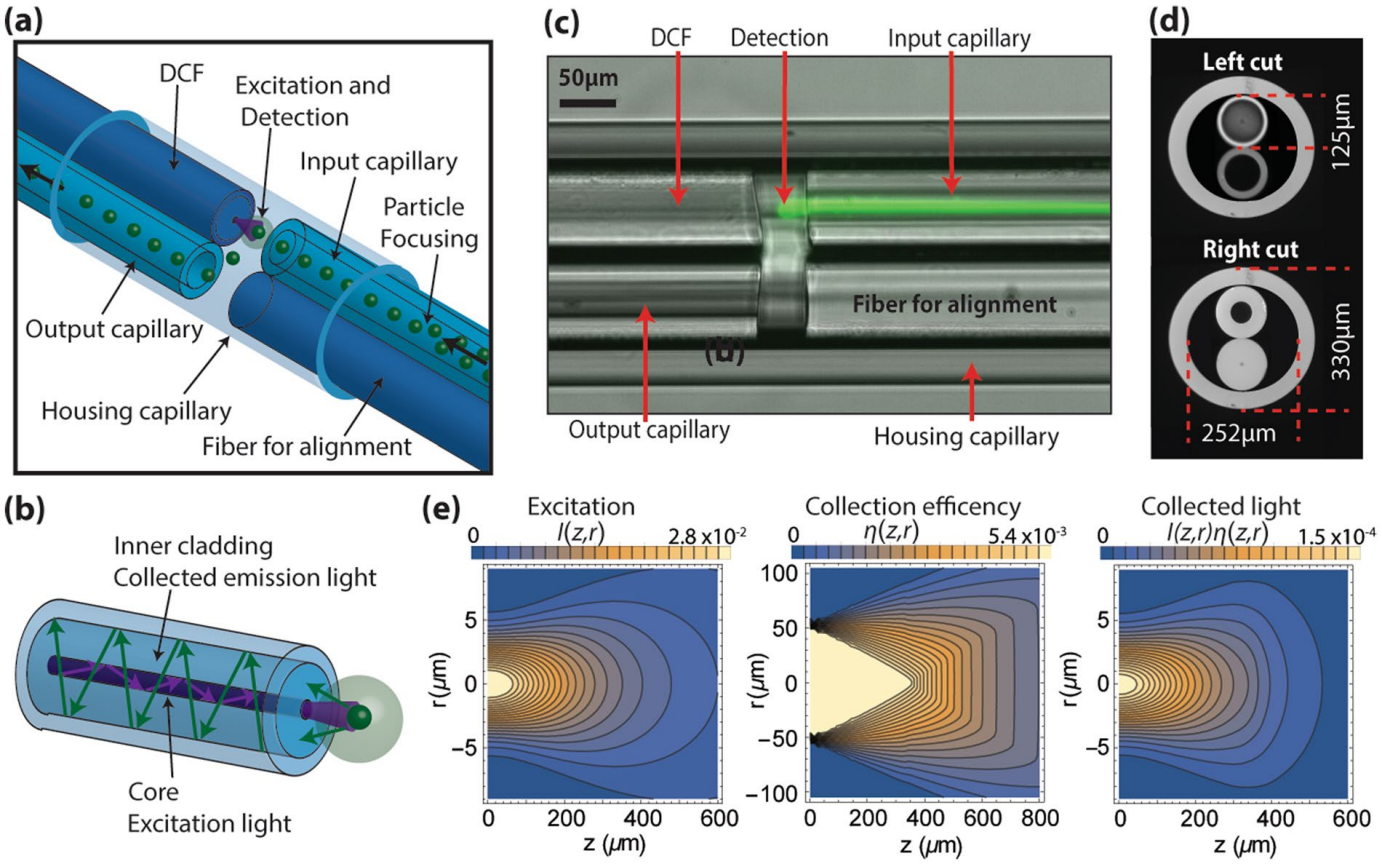
Fibre-based microflow cytometer. (**a**) Integrated detection micro-chamber; (**b**) Detection principle of the double-clad optical fibre (DCF); (**c**) Image of the micro-chamber during operation. The green light is fluorescence from particles flowing through the input capillary and excited by light from the DCF; (**d**) Cross-sectional views of the micro-chamber; (**e**) Simulated bi-dimensional map of: (left) excitation from the DCF core (9-*µm* diameter), (centre) collection efficiency of the DCF inner cladding (105-µm diameter and 0.2 NA), and (right) light collected by the DCF. An excitation wavelength 450 nm and a medium refractive index 1.33 (water) are used for the simulation.

The heart of the fibre flow cytometer is the integrated micro-chamber where analysis takes place, as illustrated schematically in Fig. 1(a). Laser light propagates in the core of a double-clad fibre^34, 35^ (DCF) to the micro-chamber and excites the fluorescent particles or cells delivered by the Input capillary one at a time. Particles are focused into a single stream at the centre of the input capillary by using Elasto-inertial microfluidics. The diameter of this capillary is chosen according to the particle size to optimize the focusing, as discussed below. In order to maximize the excitation of single particles, the DCF core diameter is small (9 µm) and precisely aligned to the centre of the input capillary.

Fluorescence and scattering from the particles are collected and guided by the large inner cladding of the same double-clad fibre to the detection system. This is schematically shown in Fig. 1(b). The use of a double-clad fibre maximizes signal collection and reduces the noise created by the reflection at the fibre end-face. This is particularly important for the scattering measurements. The diameter of the inner cladding is 105 µm and its numerical aperture (NA) is 0.2 to guarantee high collection efficiency, while the core has NA 0.12 for small divergence of the excitation light. After exposure to light, the particles exit the micro-chamber through a second capillary that has an inner diameter 90 µm (Output capillary). The length of the micro-chamber is 50 µm, which allows the flow of particles without clogging as well as efficient optical excitation and collection. An additional dummy fibre is arranged adjacent to the input capillary to facilitate alignment during manufacturing. All the fibres and capillaries above have outer diameter 125 µm, and are enclosed by a capillary (Housing Capillary) of 250/330 µm inner/outer diameter. UV-curing glue is added to the ends of the housing capillary to fix and seal the arrangement (not shown in Fig. 1(a)). The particles and cells do not interact with the glue. Figure 1(c) shows a long-exposure saturated microscope image of the device under operation. Green fluorescence is seen of particles flowing in the input capillary excited by blue light from the DCF. Figure 1(d) shows microscope images with the view from the left and right of the micro-chamber. The design of the fibre flow-cytometer is studied and optimized for light collection, as illustrated in Fig. 1(e) and in the Supplementary material note 1, where detailed calculations are presented. Excitation light exits the core into the solution, and spreads in a diffraction cone, decaying in intensity with the distance from the fibre tip^36^. A bi-dimensional map of the excitation light *I* is illustrated in Fig. 1(e, left). The inner cladding collection efficiency *η* (i.e., the fraction collected of the light emitted by a particle)^37^ is shown in Fig. 1(e, centre). For the fibre used, *η* is maximum and saturated at 5.4 × 10^−3^. As shown below, this is sufficient to detect labelled cells in an integrated fibre micro-chamber. The collected light, which is proportional to the product of the excitation intensity and the detection efficiency, is maximized for particles located on the fibre axis and close to the fibre end, as illustrated in Fig. 1(e, right). Therefore, the short distance between the input capillary and the double-clad fibre (50 µm) optimizes the collection of light and the sensitivity.

### Elasto-inertial focusing

Inertial and elasto-inertial microfluidics, using Newtonian and non-Newtonian fluid, respectively, can provide efficient single-stream particle and cell focusing that guarantees optimal sensitivity and accuracy in flow cytometry. Focusing by inertial microfluidics relies on the balance between shear-lift and wall-interaction forces^38–40^, present in fluids at Reynolds numbers ~1–100. These inertial forces cause particles to migrate to four positions in straight channels with square cross section^40^ and to an annular band in straight channels with circular cross section^41^. Single-stream focusing can be achieved at high flow rates with inertial microfluidics, but only using curved geometries such as spiral^19^ or serpentine channels^20^. In contrast, as mentioned above Elasto-inertial microfluidics can provide single-stream focusing at the centre of straight channels.

**Figure 2:**
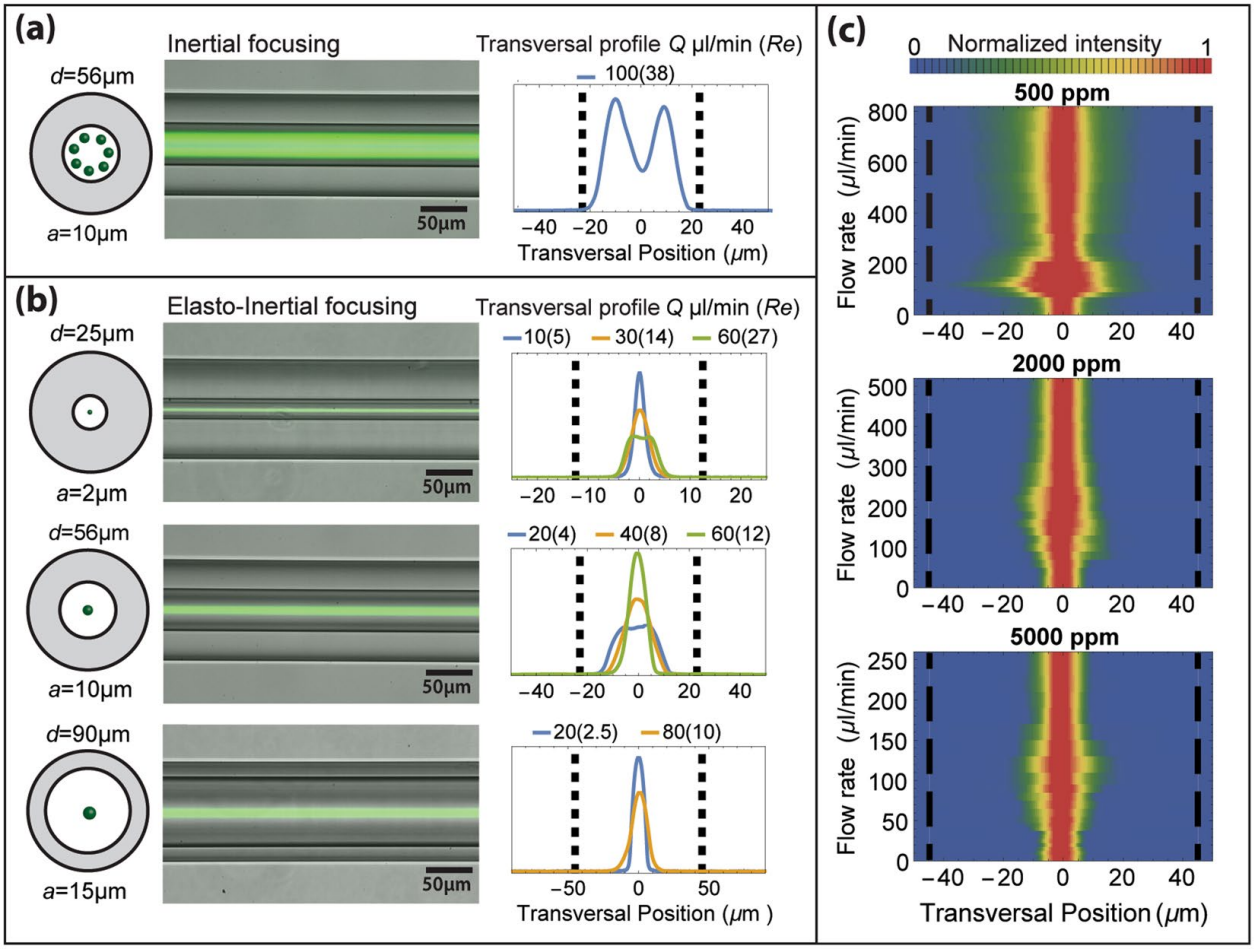
Particle focusing in capillary flow. (**a**) Inertial and (**b**) elasto-inertial focusing of particles of diameter *a* flowing in capillaries of diameter *d*. (left) schematic cross-section and long-exposure fluorescence microscopy image of focused particles, and (right) transversal profile obtained from fluorescence images indicating particle position for different flow rate (*Q*) and Reynold number (*Re*). Dashed black lines define the capillary walls; (**c**) Elasto-inertial focusing of 15-µm particles flowing in a 90-µm capillary at different flow rates for PEO concentrations of 500, 2000 and 5000 ppm. The figures consist of a contour plot made by stacking transversal profiles as a function of the flow rate.

In order to optimize the performance of the fibre microflow cytometer, inertial and elasto-inertial focusing in circular capillaries is characterized experimentally. Figure 2(a) and (b) show fluorescence microscope images of labelled particles (see Methods) flowing in circular capillaries. As expected, inertial microfluidics with PBS solution (see Methods) leads to particles being organized into an annular band at ~0.6 times the capillary radius^41^, as illustrated in Fig. 2(a). In this case, 10-µm particles flow in a 56-µm diameter capillary. Elasto-inertial microfluidics with 500 ppm PEO solution (see Methods) causes particles to focus into a single-stream at the centre of the channel. This behaviour is shown in Fig. 2(b) for 2-µm, 10-µm and 15-µm particles flowing in 25-µm, 56-µm and 90-µm diameter capillaries, respectively. The sizes of the particles are chosen to emulate those of bacteria and cells. Focusing is achieved under specific conditions^24^, which depend on the flow rate (*Q*), Reynolds number (*Re*) and ratio between particle size and channel diameter, as indicated by the transversal profiles in Fig. 2(b).

To investigate the effects of viscoelastic concentration on particle focusing, sets of experiments were performed for 15-µm particles in a 90-µm capillary for different PEO concentrations and flow rates. Stable focusing is found for concentrations from 500 ppm to 10000 ppm. Examples are given in Fig. 2(c) at 500, 2000 and 5000 ppm. Measurements for PEO concentrations 200 ppm and 10000 ppm are presented in Supplementary Figure 1. For 500 ppm, particle focusing is observed over a wide range of flow rates up to 800 µl/min, with corresponding Re up to 100. Increasing the PEO concentration improves slightly the focusing, but limits the maximum achievable flow rate, because of the relatively high pressure drop in the capillary. Furthermore, it is noted that the particles defocus partially in a limited range of flow rates, particularly for low PEO concentrations. The physics of the extended regime and the defocusing observed are still under investigation.

Based on these results, a concentration of 500 ppm is chosen for cytometry experiments since it provides a high throughput. For this concentration, stable focusing is obtained for particles of diameter from 2 µm to 24 µm, using capillaries of appropriate diameter. (See Supplementary Figure 2 for 10-µm and 24-µm particle focused in 90-µm capillary).

### Fibre microflow cytometer: characterisation with fluorescent particles

The fibre microflow cytometer is validated using labelled particles (see Methods). Two laser beams of wavelength 450 nm and 635 nm are launched into the DCF core for particle excitation. Light from the particles is collected by the inner cladding and guided to three photomultipliers, which detect scattering at 450 nm and fluorescence centred at 508 nm and 658 nm (See Methods for a detailed description of the experimental setup).

**Figure 3:**
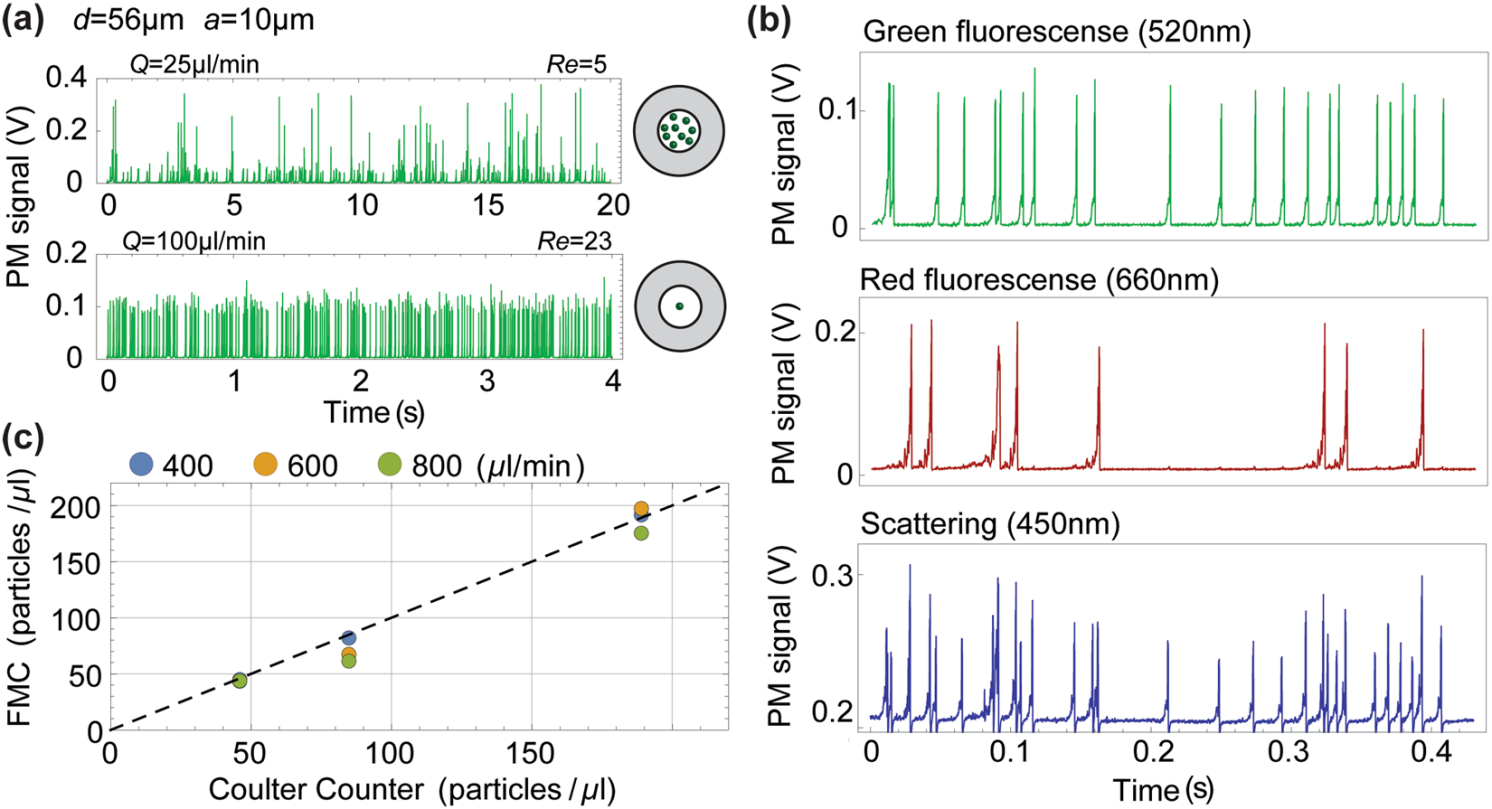
Flow cytometer characterisation. (**a**) Detection of 10-µm fluorescent particles in a 56-µm diameter capillary. Photomultiplier (PM) signal for (top) unfocused particles flowing at 25 µl/min, and (bottom) focused particles flowing at 100 µl/min; (**b**) Detection of scattering and fluorescence from a mixture of green and red 10-µm labelled particles. Green, red and blue traces represent green fluorescence centred at 508 nm, red fluorescence centred at 658 nm, and scattering at 450 nm, respectively. A total of 8003 green and 2210 red fluorescence particles are detected in 2 minutes, while the scattering events are 10113; (**c**) Comparison between number of particles measured by the fibre microflow cytometer (FMC) and by Coulter counter for flow rates of 400, 600 and 800 µl/min and three different concentrations. 15-µm particles are focused in a 90-µm capillary.

Figure 3(a) shows the effect that single-stream focusing has on particle counting. 10-µm diameter green fluorescence particles are injected into the input capillary and flow to the detection micro-chamber. The input capillary diameter is chosen to be 56 µm, which provides conditions for obtaining both unfocused and focused particle streams depending on the flow rate, as can be seen in the transversal profile of Fig. 2(b). Detection of unfocused particles, flowing 25 µl/min, is shown in Fig. 3(a, top). The amplitude of the fluorescence peaks is non-uniform, presenting a coefficient of variation (CV, ratio between standard deviation and mean) of 88%. This is attributed partly to the dependence of the collected light intensity on the particle’s position (Fig. 1(e)) and partly to multiple particles reaching the detection area simultaneously, resulting in higher amplitude counts. Single-stream focusing is achieved by increasing the flow rate to 100 µl/min, which produces uniform amplitude peaks (CV = 9%), Fig. 3, bottom).

To demonstrate the versatility of the fibre-based platform, simultaneous detection of two colour fluorescence and scattering was carried out with a mixture of 10-μm diameter green and red fluorescence particles (See Methods) flowed at 100 µl/min. As an example, Fig. 3(b) shows a time-slot of 0.4 seconds in a 2-minutes recording of the three signals produced by focused particles. There is a 99% agreement between scattering peaks and the sum of red and green fluorescence peaks.

The system accuracy and throughput is further studied by using 15-µm green fluorescent particles focused in a 90 µm capillary, allowing focusing at flow rates up to 800 µl/min. The number of particles counted with the fibre flow cytometer is compared to measurements performed with a Coulter counter (Beckman coulter, Z2). The results of three different particle concentrations, measured in triplicate, flowing at three different flow rates (400, 600 and 800 µl/min) are shown in Fig. 3(c). The linear correlation between the measurements indicates that the fibre microflow cytometer can perform accurate particle counting. The highest concentration used (200 particles/µl) defines the maximum throughput to be 2500 particles/s at a flow rate of 800 µl/min. Above this concentration, the overlap between detected peaks makes the data analysis more troublesome.

### Fibre microflow cytometer: cell counting

**Figure 4:**
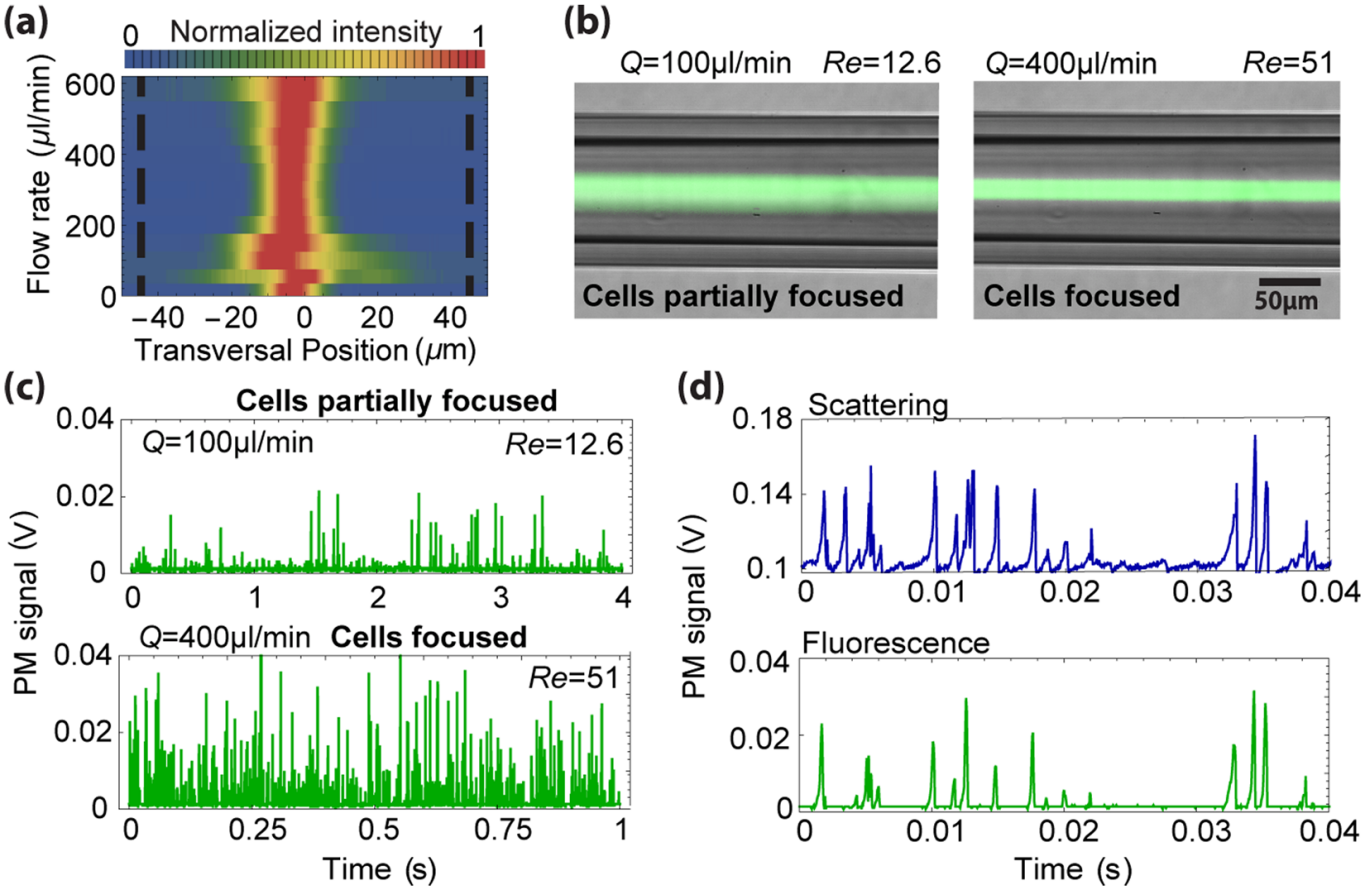
Detection of cells. (**a**) Focusing characterisation in a 90 µm capillary; (**b**) Long-exposure fluorescence microscope images of partially focused (left) and focused regimes (right); (**c**) Fluorescence signals corresponding to the images in (**b**); (**d**) Example of scattering and fluorescence over a 0.04 s interval. Data was obtained from a 1 minute recording with a total of 35484 fluorescence and 46101 scattering events.

To evaluate the system for the analysis of biological cells, cancer cell lines of ~15-µm diameter (see Methods) are counted by the fibre flow cytometer. Firstly, the focusing of cells flowing in a 90-µm capillary is characterized, Fig. 4(a). A behaviour similar to that previously observed for particles is seen. A partially focused regime, at 100 µl/min, and a focused regime, at 400 µl/min, can be identified in Fig. 4(a). The corresponding fluorescence microscope images are illustrated in Fig. 4(b). The effect that focusing has on the detection of cells is analysed by performing fluorescence detection in both regimes, Fig. 4(c). It demonstrates that single-stream focusing dramatically improves the sensitivity and enables accurate cell counting. The non-uniformity of the peaks is attributed to the nonhomogeneous labelling and size variation of cells. Finally, Fig. 4(d) shows a 0.04 seconds’ time-slot of a scattering and fluorescence measurement. In this case, the number of scattering events is higher than that of fluorescence events due to incomplete cell labelling.

## Discussion

The silica fibre based microflow cytometer presented above integrates elasto-inertial single-stream focusing of particles with scattering and fluorescence detection. It can provide peak amplitudes with a coefficient of variation 9%. The variation is attributed to minor focusing fluctuations and to non-uniform fluorescence of the particles used (nominally < 5%). Two-channel fluorescence detection of particles is performed in this work, with scattering counts overlapping fluorescence events in 99% of cases. The number of channels can be further increased by using additional optical components (couplers, filters and detectors) either in free-space optics or in fibre. Cancer cell counting is demonstrated as a first life-science application of the system. The high sensitivity obtained when cells are focused in a single stream and the ability to focus particles of 2-µm diameter suggest that the system is also capable of measuring bacteria. Additionally, the capabilities of the present device could be extended, by using high NA optical fibres for enhanced sensitivity^42^, to spectral characterization of flowing cells. For instance, this could make tracing the Raman signature of cells possible, allowing for label-free characterization.

A throughput of 2500 particle/s is achieved by exploiting elasto-inertial focusing at high flow rates (up to 800 µl/min). This is enabled by using circular cross-section channels that exclude corner effects found in rectangular geometries^27, 33^, and by using optimized PEO solutions that allow focusing at lower polymer concentrations (500 ppm). At present, the pressure drop because of the capillary resistance prevents from flowing the solution at a rate higher than 800 µl/min. Hence, the flow rate (and consequently the throughput) could be further increased by using a high-pressure pump and by optimizing the capillary length.

PEO and PVP are the most widely used elasticity enhancers and have been employed in several applications, such as bacteria^31^ and blood cells separation^26, 28, 32^. Single-stream focusing in PEO or PVP solutions has been reported only at Re < 1. In this work, stable focusing is demonstrated in PEO solution at two orders of magnitude higher Re (up to 100). The fact that there is no sign of defocusing at Re = 100, suggests that particle focusing in PEO solution could be maintained at even higher Re. This assumption is supported by a recent demonstration of particle migration in a hyaluronic acid (HA) viscoelastic fluid at extremely high Re (up to 10000)^43^, which, to the best of our knowledge, is the only additional report of high Re particle focusing in a viscoelastic fluid.

In summary, a robust, portable, simple and low-cost, fibre microflow cytometer is reported. This system solves major limitations that have hampered the implementation of flow cytometers at POC.

## Methods

### Experimental setup

**Figure 5:**
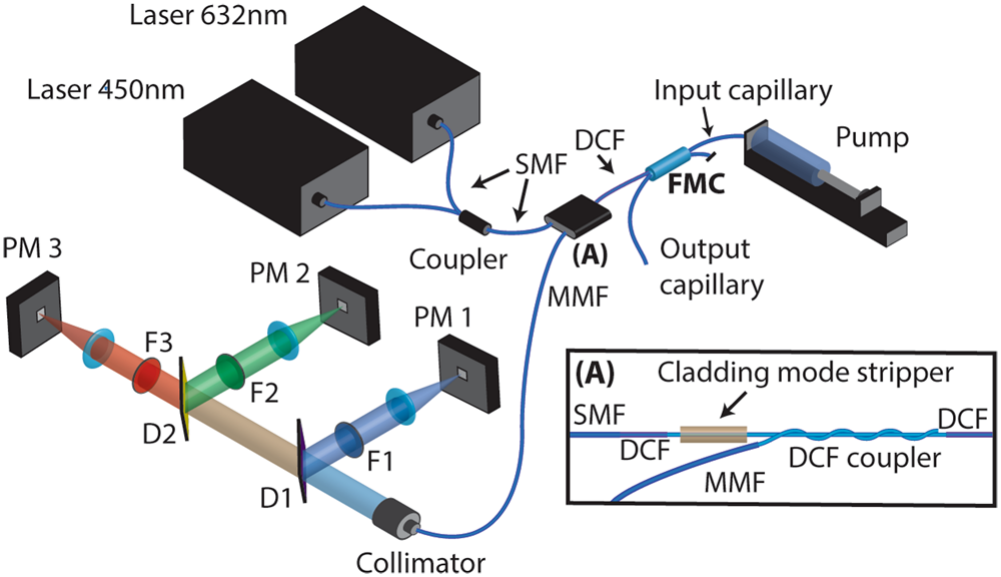
Experimental setup. SMF: single mode fibre, FMC : fibre micro-chamber (Fig. 1(a)). DCF: double-clad fibre. MMF: multi-mode fibre: F1, F2, F3: bandpass filter. D1, D2: dichroic mirror. PM 1,PM 2,PM 3 silicon photomultiplier.

The experimental setup is depicted in Fig. 5. A solution containing particles or cells is pumped to the micro-chamber through the input capillary with a syringe pump (Nemesys, Centoi Gmbh). The length of the input and output capillaries is ~10 cm. The DCF transmits the excitation light in the core to the micro-chamber and the collected emission and scattering in the inner cladding to the detection system. The light travelling in the inner cladding of the DCF is coupled out to a multimode fibre (MMF) with an efficiency ~50% using a home-made proximity coupler^34^ (DCF coupler, (A) in Fig. 5). It consists of a short section of the DCF (~5-cm long) which is etched, so that the external low-index cladding is removed and the inner cladding is exposed. The MMF (105 µm core diameter) is also etched along a few centimetres losing its cladding, and its core is wound around the etched section of the DCF. In this way, the coupler extracts to the MMF the light collected by the DCF. Excitation light from two single-mode pigtailed diode lasers at 450 nm (Thorlabs LP450-SF15) and 635 nm (Thorlabs LP635-SF8) are multiplexed using a standard fused coupler for visible light (Thorlabs FC632-50B). The output of the fused coupler, which is single-mode fibre (SMF), is spliced to the DCF. The splice is not ideal and to prevent light leaking from core into the cladding from reaching the DCF coupler, UV curing glue (refractive index = 1.51) is used as a lossy recoating along 10-cm length (cladding mode stripper, (A) in Fig. 5). The proximity coupler and the cladding mode stripper are important components to minimize return loss present in commercial fused couplers, and make possible measuring weak scattering events from particles and cells. It should be noted that the excitation light is guided in the DCF core that is single-mode at 1.5 µm wavelength and multimode in the visible, however no mode instabilities were observed. The emission light extracted by the MMF is collimated and spectrally separated in a detection system that consists of three silicon photomultipliers (Ketex PM1150). The scattered light at 450 nm is reflected by the first dichroic mirror D1 (Thorlabs DMLP490), and focused on the photomultiplier PM1. The remaining fluorescence is separated in the green (centred at 508 nm) and red channel (centred at 658 nm) by a second dichroic mirror D2 (Thorlabs DMLP605) and focused on the detectors PM2 and PM3, respectively. Light at undesired wavelengths is filtered out by colour filters F1 (Thorlabs MF445-45), F2 (Thorlabs MF525-39), and F3 (Thorlabs FEL0650).

### Fluorescent particles

Fluorescent polystyrene particles (FluoSpheres, Invitrogen and Fluoro-Max, Thermo Scientific) of diameter (fluorescence colour) 2 µm (red), 10 µm (green/red), 15 µm (green) and 24 µm (green) were used in this study. The green fluorescent particles have excitation (emission) centred at 468 nm (508 nm) and the red fluorescent particles have excitation (emission) centred at 625 nm (658 nm), respectively.

### Inertial and elasto-inertial microfluidics experiments

For the inertial microfluidics experiment, fluorescent particles were suspended in a Phosphate Buffer Saline (PBS 1x) aqueous solution containing 0.1% Tween 20. For elasto-inertial microfluidics and cytometry experiments, the particles were suspended in 500 ppm aqueous solution of Polyethylene oxide (PEO, MW = 2000000, Sigma Aldrich). For the experiments presented in Fig. 2(c), the PEO concentration was increased up to 10000 ppm. The PEO solutions were filtered with a 5-µm filter to remove debris before adding the particles or cells. A viscosity of 1.8 mPa and a density of 0.996 g/cm^−3^ are used to calculate Reynolds numbers for PEO solution at 500 ppm^44^.

### Cell preparation

HCT 116 colon cancer cells (ATCC Inc.) were cultured according to the manufacturer’s instructions, in McCoy’s 5 A media, supplemented with 2 mM Glutamine and 10% Fetal Bovine Serum, and incubated at 37 °C and 5% CO_2_. Cells were cultured until 85% confluence is obtained, harvested using trypsin-EDTA (Life technologies Inc.) for 2 minutes and mechanically dissociated by pipetting to generate a single cell suspension. The cells were passaged every 2 to 3 days. Mycoplasma contamination was tested using PlasmoTest Mycoplasma Detection Kit. Cells were pre-stained using Calcein-AM (Sigma-Aldrich) and a concentration of 1 × 10^6^ cells/ml was used.

### Imaging and analysis

An inverted microscope (Nikon Eclipse TI) with a sCMOS camera (Andoe Zyla) and LED lighting system (Lumenor Spectra X LED) was used for imaging. Micro-Manager Open Source Microscopy Software was used for microscope control and picture capturing. Images were processed by ImageJ 1.5. The PMT signals were recorded by USB oscilloscopes (Analog discovery 2.0, Digilent).

## Electronic supplementary material

Supplementary Information
