# Supplementary material for: High performance micro-flow cytometer based on optical fibres
Source: Sci Rep. 2017 Jul 17;7:5628. doi: 10.1038/s41598-017-05843-7 (PMC5514097; doi:10.1038/s41598-017-05843-7)
Supplement: Supplementary file 1 — Supplementary Information [file 41598_2017_5843_MOESM1_ESM.pdf]

High performance micro-flow cytometer based on optical fibres

S. Etcheverry[1,2*], A. Faridi,[3] H. Ramachandraiah,[3] T. Kumar[3], W. Margulis,[1,2] F. Laurell,[1] and A. Russom[3]

[1]Department of Applied Physics, KTH Royal Institute of Technology, Stockholm, Sweden.

[2]Department of Fibre Optics, RISE Acreo AB, Stockholm, Sweden

[3]Division of Proteomics and Nanobiotechnology, Science for Life Laboratory, KTH Royal Institute of Technology, Solna, Sweden

Supplementary note 1: Theoretical background for excitation and collection of light

The laser beam diverging from the core of the double-clad fiber (DCF) has a Gaussian intensity profile *I(r,z)* given by:

$$I(r,z) = \frac{2P}{\pi w(z)^2} exp\left(\frac{-(2r)^2}{w(z)^2}\right) \quad (1)$$

where P is the total power and $w(z)$ is the half width of the beam, which depends on the laser wavelength λ, the refractive index of the medium n and the core diameter d_{core},

$$w(z) = \frac{d_{core}}{2}\sqrt{1+\left(\frac{4\lambda\, z}{{d_{core}}^2 n}\right)^2} \quad (2)$$

When the laser beam excites a fluorescent particle, a portion of the fluorescence is collected by the inner cladding of the double-clad fiber and is subsequently measured. The particle can be considered as a point-source located at *(r,z)* which emits light in all directions. The fraction of the light that reaches the inner cladding is defined by the solid angle[1]:

$$\Omega_f(r,z) = 2\pi\left(1 - cos\left[tan^{-1}\left(\frac{d_{clad}}{2z}\left(cos\left(tan^{-1}\left(\frac{r}{z}\right)\right)\right)^{\frac{3}{2}}\right)\right]\right) \quad (3)$$

where d_{clad} is the diameter of the DCF inner cladding. Additionally, the numerical aperture NA of an optical fiber defines an acceptance solid angle Ω_{NA},

$$\Omega_{NA} = 2\pi\left(1 - cos\left(sin^{-1}\frac{NA}{n}\right)\right) \quad (4)$$

Light entering the fiber at angles larger than Ω_{NA} is not guided by total internal reflection and lost. The collection efficiency $\eta(r,z)$ normalized to 4π can be calculated as,

$$\eta(r,z) = \frac{Min(\Omega_f(r,z), \Omega_{NA})A(r,z)}{4\pi} \quad (5)$$

where $A(r,z)$ represents the fraction of the light encompassed by Ω_{NA} that overlaps the DCF inner cladding[1]. The collection efficiency $\eta(r,z)$ is constant and maximum for $\Omega_{NA} > \Omega_f$ and decreases with z for $\Omega_f > \Omega_{NA}$.

Supplementary figure 1

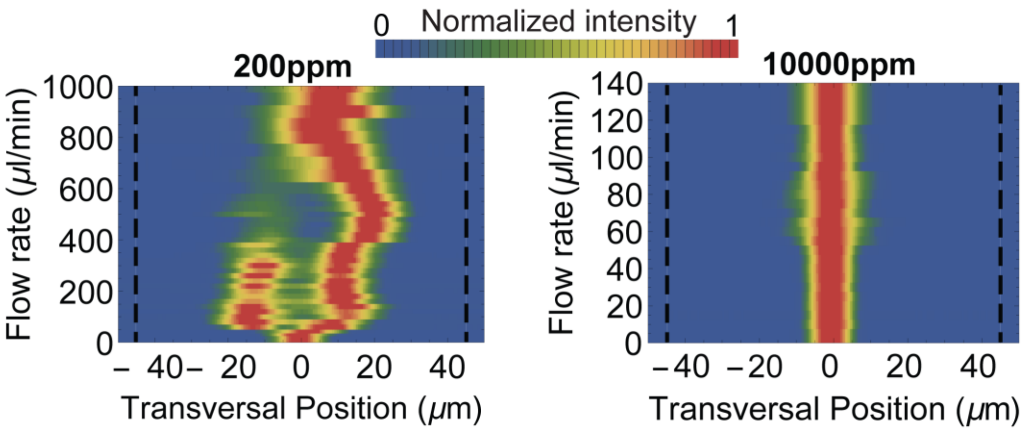

Elasto-inertial focusing of 15-μm particles flowing in a 90-μm capillary at different flow rates for PEO concentrations of 200 ppm and 10000 ppm. Dashed black lines define the capillary walls.

Supplementary figure 2

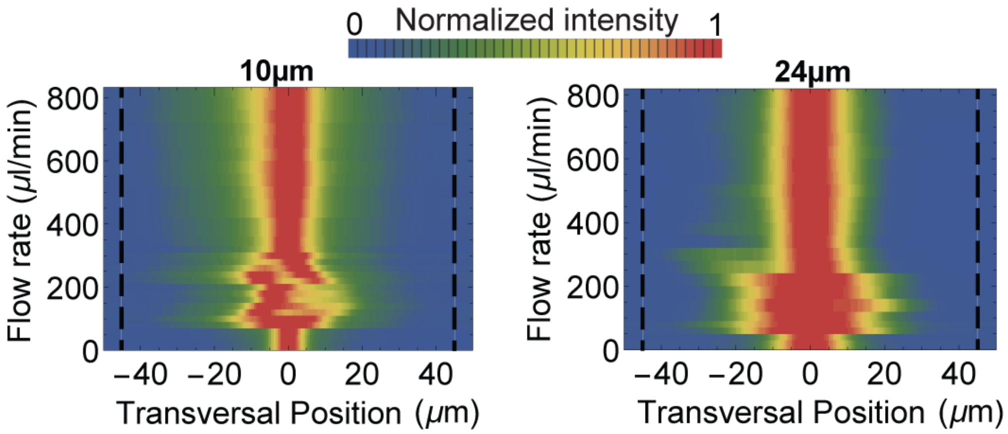

Elasto-inertial focusing of 10-µm and 24-µm particles flowing in a 90-µm capillary at different flow rates/ for PEO concentration of 500 ppm . Dashed black lines define the capillary walls.

References

1. Engelbrecht, C. J., Göbel, W. & Helmchen, F. Enhanced fluorescence signal in nonlinear microscopy through supplementary fiber-optic light collection. *Opt. Express* **17,** 6421–35 (2009).
